# Energy Metabolism and Diet

**DOI:** 10.3390/nu13061907

**Published:** 2021-06-01

**Authors:** Arie G. Nieuwenhuizen, Evert M. van Schothorst

**Affiliations:** Human and Animal Physiology, Wageningen University, 6708 WD Wageningen, The Netherlands; evert.vanschothorst@wur.nl

Energy metabolism at whole body and cellular, and even organelle (i.e., mitochondrial), level requires adequate regulation in order to maintain or improve (metabolic) health. In eukaryotic cells, mitochondria are key players in energy (ATP) production via oxidative phosphorylation. Both macro- and micronutrients potentially influence energy metabolism and mitochondrial functioning, either as substrates for (oxidative) catabolism or as essential constituents of enzymes or protein complexes involved in (mitochondrial) energy metabolism (Figure 1).

In this issue, a range of new articles are presented, and we are fortunate to have a collection of empirical preclinical and human studies to assist in the development of understanding and progress in this area of research on improving health, and, in more detail, metabolic health. The studies in this Special Issue deal with various aspects of nutrition, as summarized below:

**Energy Balance**

Focused on the topic of energy balance, Cooney and colleagues report findings of a weight loss study in ageing Irish adults with overweight and adiposity-based chronic disease [1]. Participants had dietary energy requirements prescribed on the basis of either measured resting metabolic rate (mRMR) or estimated RMR by the prediction of Miffin [1]. A similar weight loss (>5%) over the short-term period of 12 weeks was seen in these two groups, together with a reduction in blood pressure, triglycerides, and glucose, thus reducing cardiovascular disease risk factors. Cumulatively, these data further support the use of RMR, either measured or estimated, to determine energy intake during a weight loss program [1].

**Macronutrient Composition**

In recreational athletes, Terink and colleagues elegantly showed, by using a cross-over study where athletes consumed one of two diets in random order with a wash-out period of >2 weeks in between, that a low-carbohydrate, high-fat (LCHF) diet resulted in reduced workload with metabolic effects and a pronounced exercise-induced cortisol response after 2 days, when compared to a high-carbohydrate (HC) diet. Although indications of adaptation were seen after 2 weeks on the LCHF diet, work output was still lower [2].

**Specific Nutrients**

Starting with the trace element iron, amongst others involved in oxidation–reduction reactions of energy metabolism, Rineau and colleagues focused on endurance capacity and fatigue, one of the main symptoms of iron deficiency [3]. They showed that iron deficiency without anemia in mice significantly reduced endurance and activity of the respiratory chain complex I in the predominantly slow-twitch musculus soleus, but not in the musculus quadriceps. This was seen without differences in complex IV activity in both muscles. They concluded that iron deficiency without anemia results in impaired mitochondrial complex I activity in skeletal muscles with predominantly oxidative metabolism, which might explain the observed reduction of fatigue and improved physical activity when correcting iron deficiency in humans [3].

In light of the increasing number of people with obesity and associated noncommunicable diseases nutritional approaches are highly warranted to combat developments of type 2 diabetes and the spectrum of conditions ranging from increased intrahepatic accumulation of triacylglycerols (fatty liver), hepatic steatosis, steatohepatitis (NASH) and end-stage liver disease,. Previously, it has been well reported that fish oils, and more specifically, the fatty acids eicosapentaenoic acid (EPA; 20:5n-3) and docosahexaenoic acid (DHA; 22:6n-3), contribute to health benefits, including, but not limited to, nonalcoholic fatty liver disease (NAFLD; reviewed by, e.g., Chang and colleagues (Prostaglandins Leukot. Essent. Fat. Acids, 2018). In this special issue, Sistilli and colleagues [4] and Bardova and colleagues [5] show some new insights revealing the nutritional power of these fatty acids as part of fish oil triglycerides or of krill oil (and its constituents), which includes high levels of phospholipids (PL) composed of a glycerol backbone with two fatty acids (either EPA or DHA) and a phosphate group modified with simple organic molecules such as choline, ethanolamine, or serine. Sistilli et al. showed impressive antisteatotic effects in the liver by krill oil versus fish oil using an obese, insulin-resistant mouse model of exacerbated NAFLD based on high-fat feeding at thermoneutral temperature. Moreover, effects were seen in both the prevention and reversal of hepatic steatosis. This was associated with improved hepatic insulin sensitivity and high plasma adiponectin levels [4].

Bardova et al., in contrast, investigated potential additive effects by combining nutritional and pharmacological interventions, using fish oil together with a first- or second-generation antidiabetic drug, thiazolidinedione (TZD). Focusing on white adipose tissue, increased fatty acid futile cycling (triacylglycerols → free fatty acids + glycerol → triacylglycerols) supporting energy dissipation was seen as an additive beneficial effect of fish oil and TZDs, together with increased metabolic health in these diet-induced obese mice. This included reduced body weight gain, and improvements in circulating and tissue metabolites and parameters of both lipid and glucose homeostasis [5].

Together, the studies of this Special Issue provide novel detailed insights into the physiological nature of the close relationship between (nutrients) our diet, energy metabolism, and physical functioning, and confirm the importance of this relationship for maintaining good health.

## Figures and Tables

**Figure 1:**
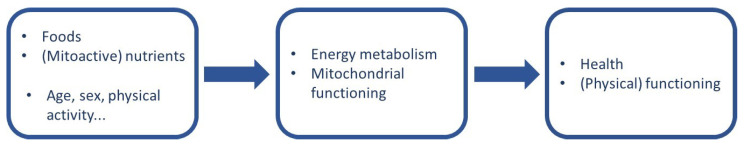
Schematic concept on the interaction between diet, energy metabolism, and health.

## Data Availability

Not applicable.

## References

[1] Cooney C., Daly E., McDonagh M., Ryan L. (2021). Evaluation of measured resting metabolic rate for dietary prescription in ageing adults with overweight and adiposity-based chronic disease. Nutrients.

[2] Terink R., Witkamp R.F., Hopman M.T.E., Siebelink E., Savelkoul H.F.J., Mensink M. (2021). A 2 week cross-over intervention with a low carbohydrate, high fat diet compared to a high carbohydrate diet attenuates exercise-induced cortisol response, but not the reduction of exercise capacity, in recreational athletes. Nutrients.

[3] Rineau E., Gueguen N., Procaccio V., Geneviève F., Reynier P., Henrion D., Lasocki S. (2021). Iron deficiency without anemia decreases physical endurance and mitochondrial complex i activity of oxidative skeletal muscle in the mouse. Nutrients.

[4] Sistilli G., Kalendova V., Cajka T., Irodenko I., Bardova K., Oseeva M., Zacek P., Kroupova P., Horakova O., Lackner K. (2021). Krill oil supplementation reduces exacerbated hepatic steatosis induced by thermoneutral housing in mice with diet-induced obesity. Nutrients.

[5] Bardova K., Funda J., Pohl R., Cajka T., Hensler M., Kuda O., Janovska P., Adamcova K., Irodenko I., Lenkova L. (2020). Additive effects of omega-3 fatty acids and thiazolidinediones in mice fed a high-fat diet: Triacylglycerol/fatty acid cycling in adipose tissue. Nutrients.

